# Cytoplasmic Incompatibility as a Means of Controlling *Culex pipiens quinquefasciatus* Mosquito in the Islands of the South-Western Indian Ocean

**DOI:** 10.1371/journal.pntd.0001440

**Published:** 2011-12-20

**Authors:** Célestine M. Atyame, Nicole Pasteur, Emilie Dumas, Pablo Tortosa, Michaël Luciano Tantely, Nicolas Pocquet, Séverine Licciardi, Ambicadutt Bheecarry, Betty Zumbo, Mylène Weill, Olivier Duron

**Affiliations:** 1 Institut des Sciences de l'Evolution, CNRS UMR 5554, Université Montpellier 2, Montpellier, France; 2 Centre de Recherche et de Veille sur les Maladies Émergentes dans l'Océan Indien (CRVOI), Ste Clotilde, France; 3 Fédération de Recherche Environnement, Biodiversité et Santé, Université de La Réunion, Ste Clotilde, France; 4 Department of Entomology, Faculty of Science, University of Antananarivo, Antananarivo, Madagascar; 5 Agence Régionale de Santé (ARS) Océan Indien, Délégation de l'Ile de Mayotte, Mamoudzou, France; 6 Groupement d'Intérêt Public Cyclotron Réunion Océan Indien (GIP CYROI), CIRAD UMR 15, Ste Clotilde, France; 7 Vector Biology and Control Division, Ministry of Health and Quality of Life, Port Louis, Mauritius; Johns Hopkins Bloomberg School of Public Health, United States of America

## Abstract

The use of the bacterium *Wolbachia* is an attractive alternative method to control vector populations. In mosquitoes, as in members of the *Culex pipiens* complex, *Wolbachia* induces a form of embryonic lethality called cytoplasmic incompatibility, a sperm-egg incompatibility occurring when infected males mate either with uninfected females or with females infected with incompatible *Wolbachia* strain(s). Here we explore the feasibility of the Incompatible Insect Technique (IIT), a species-specific control approach in which field females are sterilized by inundative releases of incompatible males. We show that the *Wolbachia w*Pip(Is) strain, naturally infecting *Cx. p. pipiens* mosquitoes from Turkey, is a good candidate to control *Cx. p. quinquefasciatus* populations on four islands of the south-western Indian Ocean (La Réunion, Mauritius, Grande Glorieuse and Mayotte). The *w*Pip(Is) strain was introduced into the nuclear background of *Cx. p. quinquefasciatus* mosquitoes from La Réunion, leading to the LR[*w*Pip(Is)] line. Total embryonic lethality was observed in crosses between LR[*w*Pip(Is)] males and all tested field females from the four islands. Interestingly, most crosses involving LR[*w*Pip(Is)] females and field males were also incompatible, which is expected to reduce the impact of any accidental release of LR[*w*Pip(Is)] females. Cage experiments demonstrate that LR[*w*Pip(Is)] males are equally competitive with La Réunion males resulting in demographic crash when LR[*w*Pip(Is)] males were introduced into La Réunion laboratory cages. These results, together with the geographic isolation of the four south-western Indian Ocean islands and their limited land area, support the feasibility of an IIT program using LR[*w*Pip(Is)] males and stimulate the implementation of field tests for a *Cx. p. quinquefasciatus* control strategy on these islands.

## Author Summary

Mosquitoes of the *Culex pipiens* complex are important vectors of human pathogens including filarial parasites and many currently expanding arboviruses. The absence of effective vaccines and the evolution of insecticide resistance stress the urgent need for the development of novel control strategies. One strategy that is receiving increasing attention is based upon the use of the intracellular bacteria *Wolbachia*, which induce a form of sterility known as cytoplasmic incompatibility in mosquitoes. Here, we show that a *Wolbachia* strain, named *w*Pip(Is) and naturally infecting *Cx. p. pipiens* from Turkey, can be used in the Incompatible Insect Technique (IIT) to sterilize *Cx. p. quinquefasciatus* females from several islands of the southwestern Indian Ocean (SWIO). The *w*Pip(Is) strain was introduced into SWIO *Cx. p. quinquefasciatus* nuclear background leading to the LR[*w*Pip(Is)] line. Males from this latter line were found to sterilize all wild females tested, and no difference in mating competition was observed between LR[*w*Pip(Is)] and wild males. These results encourage the development of an IIT program based on the *w*Pip(Is) strain to control mosquito populations in the SWIO.

## Introduction

The last few years have witnessed an increasing interest in the alpha-proteobacterium *Wolbachia* (Rickettsiales) for the biological control of insect pest populations [for reviews see [1]–[5]. *Wolbachia* is the most common intracellular bacterium yet described [6], [7], present in more than 65% of insect species and found in all major insect families [8]. Some medically important mosquitoes are naturally infected by *Wolbachia*, such as the common house mosquito *Culex pipiens* [9], [10] and the Asian tiger mosquito *Aedes albopictus* [11], or can otherwise be artificially infected, such as the yellow fever mosquito *Ae. aegypti* [12]–[14].

*Wolbachia* is vertically inherited from a female host to its progeny through the egg cytoplasm, males being a dead end in terms of transmission [4], [15]. *Wolbachia* is usually termed a ‘reproductive parasite’ in the sense that it optimizes its transmission by manipulating its host's reproductive biology [15], [16]. In mosquitoes, *Wolbachia* induces a form of embryonic death called cytoplasmic incompatibility (CI) [9]. This phenomenon results from sperm-egg incompatibility occurring when *Wolbachia*-infected males mate with uninfected females or females infected with an incompatible *Wolbachia* strain [17]. Therefore, CI has been investigated as a mechanism to control field populations [1], [18], [19], [20]–[22], or to drive transgenes into field populations [2], [3], [10], [23]. In addition, recent investigations showed that *Wolbachia* can affect virus transmission both by reducing the lifespan of the infected vector and by interfering with the arthropod-borne parasite [14], [24]–[26].

Mosquitoes of the *Cx. pipiens* complex are of special interest for *Wolbachia*-based control strategies. The most common members of the complex are the subspecies *Cx. p. quinquefasciatus* (Say) and *Cx. p. pipiens* (Linnaeus) (also considered as true species, depending on the authors), representing the southern and northern mosquito populations, which are ubiquitous in tropical and temperate regions, respectively [27]. This mosquito is the main vector of lymphatic filarial in Comoros and Madagascar [28] as well as a known vector for many arboviruses worldwide [29]. This is the case, for example, of the West Nile Virus (WNV), recrudescent in Mediterranean countries [30], [31] and in the United States where thousands of cases have been identified in the last decade [32], [33]. This species also transmits the Rift Valley Fever (RVF) virus, currently expanding in the Indian Ocean [34], [35].

Members of the *Cx. pipiens* complex are naturally infected with different *Wolbachia* strains, referred as *w*Pip strains. The prevalence is high in natural populations with *w*Pip infections near to fixation [10], [36], [37]. Recent multi-loci typing approaches revealed that the *w*Pip strains cluster into five distinct phylogenetic groups (referred as *w*Pip-I to V) which form a robust monophyletic clade within the B group of *Wolbachia* [38]. The *Cx. pipiens* complex exhibits the largest variation of CI crossing types observed in arthropods thus far [39]–[43]. When *Cx. pipiens* individuals are infected by different *Wolbachia* strains (here arbitrarily named *w*Pip(1) and *w*Pip(2)), their crosses can be (a) compatible and produce viable offspring; (b) incompatible in both directions and produce infertile eggs (a phenomenon called bidirectional CI); or (c) incompatible in one direction only (unidirectional CI, e.g. the cross between *w*Pip(1) males and *w*Pip(2) females is incompatible, while the reciprocal cross is compatible). The presence of incompatible *w*Pip infections in the *Cx. pipiens* system makes unnecessary the artificial introduction of exogenous *Wolbachia* strains, and encourages the development of a *Wolbachia*-based control strategy.

Here, we examined the feasibility of an ‘Incompatible Insect Technique’ (IIT) strategy targeting *Cx. pipiens* natural populations. IIT derives from the ‘Sterile Insect technique’ (SIT) notably used in the control of the New World screwworm *Cochliomyia hominivorax* [44]. In both SIT and IIT, mating of released sterilizing males with native females leads to a decrease in the females' reproductive potential and ultimately, if males are released in sufficient numbers over a sufficient period of time, to the local elimination or eradication of the pest population [3], [20], [22], [45]. In the SIT program, males are sterilized with irradiation or chemicals, which might weaken the fitness of sterilized insects, making them less competitive than field males for mating [46], [47]. In the IIT strategy, *Cx. pipiens* males are infected by a *w*Pip strain incompatible with the *w*Pip strain(s) infecting field females. In this case, the released incompatible males are not expected to suffer any reduction in mating when competing with field males. The IIT strategy has been successfully applied in a field trial assay targeting *Cx. pipiens* populations in Burma [48] as well as in cage experiments with the Polynesian tiger mosquito *Ae. polynesiensis* [49] and the medfly *Ceratitis capitata* [19].

We focused on natural populations of *Cx. p. quinquefasciatus* collected on five islands in the south-western Indian Ocean (SWIO): La Réunion, Mauritius, Mayotte, Madagascar and Grande Glorieuse. Prior studies have demonstrated that *Cx. p. quinquefasciatus* lines from La Réunion are infected with closely related *w*Pip strains which express complete CI (ca. 100% embryo mortality) with *Cx. pipiens* lines from distant geographic areas and infected by genetically different *w*Pip strains [43]. The *Cx. p. pipiens* Is line from Turkey, infected by the *w*Pip(Is) strain, is of particular interest: all crosses between Is males and females from La Réunion are incompatible and almost all reciprocal crosses are incompatible as well [43]. This complete bidirectional CI makes the *w*Pip(Is) strain a good candidate for an IIT program.

In this study, we obtained robust data that encourage the use of *w*Pip(Is)-infected *Cx. p. quinquefasciatus* males in an IIT program which could be implemented on the SWIO islands. First, the regional genetic diversity of *w*Pip infections is low as all identified *w*Pip strains belong to the *w*Pip-I group; this indicates that immigration of mosquitoes into the controlled area is unlikely to introduce a new *w*Pip strain compatible with *w*Pip(Is)-infected males. Second, the *w*Pip(Is) strain, from the *w*Pip- IV group, was introduced into the nuclear background of *Cx. p. quinquefasciatus* mosquitoes, leading to a line (LR[*w*Pip(Is)]) expressing complete CI with wild females sampled from all 5 SWIO Islands. Last, CI properties expressed by this line are optimal as (i) there is no effect of males ageing on CI expression, (ii) LR[*w*Pip(Is)] males show similar body size and longevity as males from La Réunion Island, suggesting good competitiveness of incompatible males *vs.* wild males, which was further confirmed in cage confrontations and (iii) LR[*w*Pip(Is)] mosquitoes are mainly bidirectionally incompatible with La Réunion, Mauritius, Mayotte and Grande Glorieuse field mosquitoes: this lowers the risk of *Wolbachia* replacement possibly induced by accidental releases of LR[*w*Pip(Is)] females.

## Materials and Methods

### Mosquito collections

**Figure 1:**
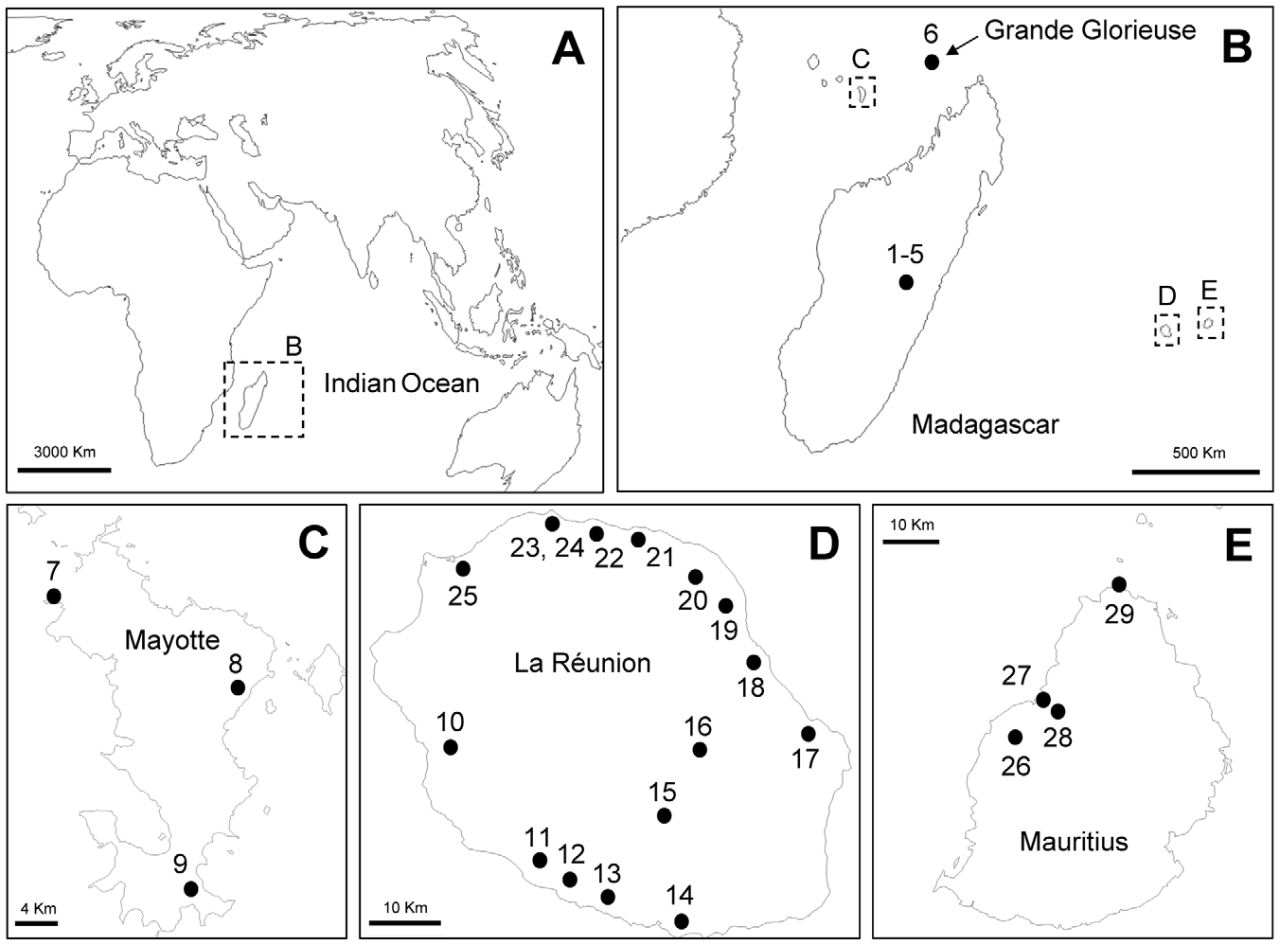
Sample site locations. A: location of the study area; B, Madagascar and surrounding islands, including Grande Glorieuse; C, Mayotte; D, La Réunion; E, Mauritius. Populations are numbered from 1 to 29. Numbers correspond to those in Table 1.

Two laboratory lines of *Cx. pipiens* mosquitoes naturally infected by *Wolbachia* were used in the experiments: the isofemale line Is, a *Cx. p. pipiens* line from Turkey infected by the *w*Pip(Is) strain, and the *Cx. p. quinquefasciatus* LR line, infected by the *w*Pip(LR) strain, and established from several hundred field-caught larvae in La Réunion island (Table 1 and Figure 1). In addition, one uninfected line, LR-TC, was generated by curing *Wolbachia* of mosquitoes from the LR line with antibiotic, following the protocol described in [50]. Briefly, ca. 5,000 LR larvae were reared for three generations in a solution containing tetracycline hydrochloride at concentrations of 10^−4^, 2×10^−4^ and 4×10^−4^ M for the first, second and third-instar larvae, respectively. Mosquitoes from LR-TC were next reared for at least two generations in the absence of tetracycline before experiments, to prevent any possible side-effects of the treatment.

**Table 1:** Mosquito collections.

Mosquito collections	*Culex pipiens* taxon	Origin	Number of screened field specimens^d^	Year of collection	Reference
Laboratory lines					
Istanbul (Is)	*pipiens*	Istanbul (Turkey)	_	2003	[34]
LR	*quinquefasciatus*	Etang Salé, Saint Pierre and Saint Louis (La Réunion)^a^	_	2009	This study
LR-TC	*quinquefasciatus*	derived from LR^b^	_	_	This study
LR[*w*Pip(Is)]	*_*	derived from LR and Is^c^	_	_	This study
Natural populations					
#1, Tana1	*quinquefasciatus*	Antananarivo (Madagascar)	23	2010	This study
#2, Tana2	*quinquefasciatus*	Antananarivo (Madagascar)	22	2010	This study
#3, Itaosy1	*quinquefasciatus*	Antananarivo (Madagascar)	20	2010	This study
#4, Itaosy2	*quinquefasciatus*	Antananarivo (Madagascar)	20	2010	This study
#5, Mada	*quinquefasciatus*	Antananarivo (Madagascar)	20	2011	This study
#6, Grande Glorieuse	*quinquefasciatus*	Grande Glorieuse	24	2011	This study
#7, Acoua	*quinquefasciatus*	Acoua (Mayotte)	24	2011	This study
#8, Tsoundzou	*quinquefasciatus*	Tsoundzou (Mayotte)	21	2010	This study
#9, M'Tsamoudou	*quinquefasciatus*	M'Tsamoudou (Mayotte)	24	2011	This study
#10, Saint Leu	*quinquefasciatus*	Saint Leu (La Réunion)	24	2007	[41]
#11, Etang Salé	*quinquefasciatus*	Etang Salé (La Réunion)	24	2009	[41]
#12, Saint Louis	*quinquefasciatus*	Saint Louis (La Réunion)	23	2009	[41]
#13, Saint Pierre	*quinquefasciatus*	Saint Pierre (La Réunion)	24	2009	[41]
#14, Saint Joseph	*quinquefasciatus*	Saint Joseph (La Réunion)	19	2007	[41]
#15, Plaine des Cafres	*quinquefasciatus*	Plaine des Cafres (La Réunion)	24	2009	[41]
#16, Plaine des Palmistes	*quinquefasciatus*	Plaine des Palmistes (La Réunion)	22	2007	[41]
#17, Sainte Rose	*quinquefasciatus*	Sainte Rose (La Réunion)	24	2007	[41]
#18, Saint Benoît	*quinquefasciatus*	Saint Benoît (La Réunion)	24	2007	[41]
#19, Bras Panon	*quinquefasciatus*	Bras Panon (La Réunion)	32	2007	[41]
#20, Saint André	*quinquefasciatus*	Saint André (La Réunion)	24	2007	[41]
#21, Sainte Suzanne	*quinquefasciatus*	Sainte Suzanne (La Réunion)	24	2007	[41]
#22, Sainte Marie	*quinquefasciatus*	Sainte Marie (La Réunion)	24	2009	[41]
#23, Saint Denis	*quinquefasciatus*	Saint Denis (La Réunion)	24	2007	[41]
#24, Samuel	*quinquefasciatus*	Saint Denis (La Réunion)	24	2010	[41]
#25, La Possession	*quinquefasciatus*	La Possession (La Réunion)	24	2007	[41]
#26, Beau Bassin	*quinquefasciatus*	Beau Bassin – Rose Hill (Mauritius)	42	2010	This study
#27, Salines	*quinquefasciatus*	Les Salines (Mauritius)	7	2010	This study
#28, Port Louis	*quinquefasciatus*	Port Louis (Mauritius)	19	2010	This study
#29, Cap Malheureux	*quinquefasciatus*	Cap Malheureux (Mauritius)	23	2010	This study

a , the LR line was established from several hundred of field-caught larvae from three natural populations, i.e. Etang Salé, Saint Pierre and Saint Louis.

b , LR-TC is *Wolbachia*-uninfected line generated by antibiotic exposure of specimens from the LR line.

c , the LR[*w*Pip(Is)] line combined the Is cytoplasm, including the *w*Pip(Is) strain, and the LR nuclear genome.

d , number of field specimens examined for *w*Pip genetic diversity.

Field *Cx. p. quinquefasciatus* larvae and pupae were collected during the summers 2007–2011 in 29 natural breeding sites on five islands of the Indian Ocean: La Réunion (16 populations), Mauritius (four populations), Mayotte (three populations) Madagascar (five populations) and Grande Glorieuse (one population) (Table 1 and Figure 1). Specimens were brought to the laboratory for emergence and identification. Individuals were either directly stored in 70% EtOH for molecular analyses or kept alive for crossing experiments. All mosquitoes were reared in 65 dm^3^ cages kept at ca. 25±2°C with 12 h/12 h light/dark cycle. Larvae were fed *ad libitum* with a mixture of shrimp powder and rabbit pellets, and adults with a honey solution.

### Molecular typing

Mosquito DNA was extracted using a CetylTrimethylAmmonium Bromide (CTAB) protocol [51]. The *w*Pip infections were characterized through the analysis of one *Wolbachia* marker, the ankyrin domains encoding gene, *ank*2 [52] (primers are listed in Table S1). This marker differentiated *w*Pip strains from groups I and IV on the basis of the size of the PCR amplified fragments: 313 bp and 511 bp fragment for group I and IV, respectively. For field samples, the *ank2* PCR products from two specimens per sample site were sequenced to confirm their identity with La Réunion *ank2* allele [Genbank AM397068; [43]].

The examination of the mosquito nuclear genome was assessed by PCR/RFLP tests based on *Cx. pipiens ace-2* and *Ester^2^* genes (primers are in Table S1). The *ace-2* gene is located on chromosome I and encodes acetylcholinesterase 2 (AChE2) [53]. The *Ester^2^* gene is located on chromosome II and encodes a carboxylester hydrolase [54]. A PCR/RFLP test on *ace-2* using the *ScaI* restriction enzyme (37°C, 3 hours; see [55]) allows the discrimination between the Is (two fragments: 230 and 470 bp) and the LR (three fragments: 120, 230 and 350 bp) nuclear genomes. We developed a PCR/RFLP test on *Ester^2^* using the *AvaII* enzyme (37°C, 3 hours) that also generated different restriction fragments for the Is (three fragments: 37, 519 and 544 bp) and LR (four fragments: 91, 176, 313 and 520 bp) nuclear genomes.

All PCRs were performed with ca. 20 ng of genomic DNA solution in a 40 µl final volume reaction for 35 cycles (94°C, 5 min; 94°C, 30 sec; 52°C, 30 sec; 72°C, 1 min). Direct sequencing of PCR products was performed on an ABI Prism 3130 sequencer using the BigDye Terminator Kit (Applied Biosystems) after purification with the QIAquick gel extraction kit (QIAGEN, Valencia, CA). Sequence alignment and analyses were done using MEGA software [56].

### Backcrossing

The cytoplasm of the Is line, including the *w*Pip(Is) strain, was introduced into the LR nuclear background through eight generations of backcrossing, a procedure that should result in at least 99% genome replacement of the Is line by the LR nuclear genome. A first cross was performed using 200 virgin Is females and 250 LR-TC males. For the following generations, 200 hybrid females were backcrossed with 250 LR-TC males. Using this protocol, we obtained the LR[*w*Pip(Is)] line which carries the LR nuclear genome and the *w*Pip(Is) strain.

### Crossing experiments

We examined the crossing relationships between mosquito lines through crossing experiments. Mass crosses were carried out using 35–200 two-day-old males and an equivalent number of females that had been individually separated at the pupal stage (age was assessed from the emergence of adults; day 0 = emergence). We also tested the effect of male aging on CI by comparing crossing relationships of young males (two-day-old) to that of older males (24-day-old). For all crosses, females were allowed to blood feed 5 days after caging. Egg-rafts were collected and stored separately until hatching at 25°C±2°C. Hatching rates (HR) were scored 72 h after egg-raft collection to determine the CI phenotype. All unhatched egg-rafts were checked for fertilization through observation of embryonic development following the procedure of [57].

### Male performance

The longevity of the LR[*w*Pip(Is)] and LR males was compared. We obtained males from larvae reared in standardized laboratory conditions at ca. 25°C±2°C. For each line, three containers containing 300 first-instar larvae with 1 L of water were set up. The water of each container was changed every two days and food provided *ad libitum*. Pupae were randomly sampled from the three containers to minimize possible rearing bias. Pupae were placed separately in 5 mL vials for emergence. Freshly-emerged males were kept in their vials until they died, and mortality was checked twice a day. No food was provided to the adults but they had access to the water in their tube. Survival data were fitted to the Cox proportional hazards models (*coxph*, survival package) [58] and a ratio for each line was estimated as their instantaneous risk of death relative to each other. These analyses were performed using R software (www.r-project.org). One posterior leg was removed on dead specimens and the tibia was measured with a micrometer (NIKON Digital Counter CM-6S).

Four cages were set up to compare the mating performance of both LR[*w*Pip(Is)] and LR males. Each cage contained an equal number of two-day-old virgin LR females and LR males (1∶1), as well as different numbers of two-day-old virgin LR[*w*Pip(Is)] males so that different ratios of the three types of mosquitoes could be tested (1∶1∶0, 1∶1∶1, 1∶1∶5 and 1∶1∶10). Thus the total number of adults for each of these confrontations was 200, 300, 350 and 600, respectively. For each confrontation, all the mosquitoes were introduced into the cage at the same time. Females were allowed to blood feed five days after caging and their egg-rafts were collected daily to score HR. To assess the stability of the expression of CI over the mosquito lifespan, a second blood meal was given to females 15 days after the first one, and new collections of egg-rafts were then made.

## Results

### Only one *w*Pip group is present in the south-western Indian Ocean islands

We first examined the genetic diversity of *w*Pip strains found in natural populations of *Cx. p. quinquefasciatus* from La Réunion, Mauritius, Mayotte, Madagascar and Grande Glorieuse. The main purpose of this investigation was to assess the possibility of controlling mosquito populations in each of these four islands with *w*Pip(Is)-infected males.

We examined 650 *Cx. p. quinquefasciatus* field specimens from 29 populations: La Réunion (16 populations, n = 384 individuals), Mauritius (4 populations, n = 91 individuals), Mayotte (3 populations, n = 69 individuals), Madagascar (5 populations, n = 105 individuals) and Grande Glorieuse (1 population, n = 24) (Table 1 and Figure 1). The genotyping of *w*Pip infections in these samples was performed using only the *ank2* gene which was recently shown to discriminate *w*Pip strains into five distinct phylogenetic groups (referred as *w*Pip-I to *w*Pip-V) [38].

PCR assays using *ank2* indicated the occurrence of *w*Pip infection in all *Cx. p. quinquefasciatus* field specimens, as observed in other geographic areas for this species [10], [36], [37], and all shared the same *ank2* allele as indicated by the length of *ank*2 PCR products (313 bp). This similarity was further confirmed by sequencing the *ank2* gene of two individuals per population from Mauritius, Mayotte, Madagascar and Grande Glorieuse.

All sequences were found to be strictly identical to that found in the *w*Pip strains infecting all 10 laboratory isofemale lines from La Réunion and to other *w*Pip strains belonging to the *w*Pip-I group [38]. This result shows that *w*Pip strains from La Réunion, Mauritius, Mayotte, Madagascar and Grande Glorieuse are genetically closely related and are genetically different from the *w*Pip(Is) strain belonging to the *w*Pip-IV group.

### Establishment of the LR[*w*Pip(Is)] line

Males from the Is line belong to *Cx. p. pipiens* subspecies and may not be optimally adapted to the tropical environment of the Indian Ocean where *Cx. p. quinquefasciatus* is found. More specifically the two subspecies are known to differ by behavioral and physiological characters including mating behavior [27]. To circumvent this problem, we introduced the *w*Pip(Is) strain into the *Cx. p. quinquefasciatus* nuclear background from La Réunion. First a LR line was established from a large number (>5,000) of field-caught *Cx. p. quinquefasciatus* from three localities of La Réunion in order to have a good representation of the local genetic diversity. This line was then cured of its *Wolbachia* by tetracycline treatment of larvae during three generations (LR-TC line). Finally *w*Pip(Is) from the Is line was introduced into the nuclear background of the LR-TC line by successive backcrossing. The LR[*w*Pip(Is)] line thus created shares the same nuclear genetic background as the LR line but is infected by the *w*Pip(Is) strain (Figure S1). This was verified by PCR/RFLP tests on *ace-2* and *Ester^2^ Cx. pipiens* nuclear genes (Figure S2A and S2B) and by analyzing the allelic profiles of the *ank2* gene of the infecting *Wolbachia* (Figure S2C).

Crossing experiments between LR[*w*Pip(Is)] and Is lines were conducted to check that *Cx. p. quinquefasciatus* nuclear background has not altered the CI phenotype of the *w*Pip(Is) strain. This aspect needs to be investigated since the host nuclear genome has been reported to affect the penetration of the CI phenotype induced by a *Wolbachia* strain [59]–[61]. Our data show that both lines behave similarly: LR[*w*Pip(Is)] and Is showed bidirectional CI with LR while LR[*w*Pip(Is)] and Is were mutually compatible (Table 2). The intensity of CI was very high, with 98–100% of the embryos that did not hatch in incompatible crosses. In addition, crosses between infected and uninfected lines showed unidirectional CI: males from all infected lines (LR[*w*Pip(Is)], Is and LR ) induced complete CI (100% embryo mortality) when crossed with uninfected females (LR-TC), the reverse crosses (i.e. uninfected males and infected females) being always compatible. Overall, no significant difference of hatching rate (HR) was found when the LR[*w*Pip(Is)] and Is lines were compared (Wilcoxon test; all *P*>0.14). This shows that the CI phenotype of the *w*Pip(Is) strain was not altered by the LR genetic background, and that the CI phenotype is controlled by the *w*Pip infection rather than by nuclear genes, which is in accordance with most studies involving species of the *Cx. pipiens* complex [43], [62].

**Table 2:** Comparisons of CI properties of different mosquito lines.

Females	Males			
	LR[*w*Pip(Is)]	Is	LR	LR-TC
LR[*w*Pip(Is)]	0.919±0.027 (2298; 14)^a^	0.967±0.010 (1405; 12)^a^	0.000±0.000 (>6000; 47)^b^	0.915±0.070 (2953; 19)^a^
Is	0.958±0.016 (1414; 12)^a^	0.951±0.061 (1402; 10)^a^	0.021±0.021 (>6000; 47)^b^	n.d.
LR	0.000±0.000 (>3500; 31)^b^	0.000±0.000 (>8000; 68)^b^	0.930±0.031 (1423; 10)^a^	n.d.
LR-TC	0.000±0.000 (>2000; 21)^b^	0.000±0.000 (>1200; 15)^b^	0.000±0.000 (>3500; 31)^b^	0.905±0.028 (1423; 10)^a^

LR[*w*Pip(Is)] and Is lines were both infected by the *w*Pip(Is) strain but had different nuclear genomes. LR-TC is a *Wolbachia*-uninfected line derived from the LR line. For each cross, mean hatching rate ± standard error, number of eggs and egg-rafts are reported.

a and b represent statistical groups (Wilcoxon two sided-test with Bonferonni's adjustment for multiple comparisons); n.d., not determined.

The effect of male ageing on CI intensity was also tested as, in a few host species including some mosquitoes, CI intensity has been shown to decrease with male aging [63]–[67]. Such an effect could impede the use of LR[*w*Pip(Is)] males to sterilize field females. To investigate this aspect, we crossed two-day and 24-day old LR[*w*Pip(Is)] males with two-day old LR females. No viable embryo was obtained in incompatible crosses with both young and old LR[*w*Pip(Is)] males (Table 3). Thus CI is expressed with the same intensity throughout the LR[*w*Pip(Is)] males' lifespan, a result also observed in diverse *Cx. pipiens* laboratory lines [10], [68].

**Table 3:** Effect of LR[*w*Pip(Is)] males ageing on CI phenotype.

Crosses	Hatching rate	
	2-day old males	24-day old males
♂LR[*w*Pip(Is)]×♀ LR[*w*Pip(Is)]	0.919±0.027 (2298; 14)^a^ †	0.940±0.030 (900; 8)^a^
♂LR[*w*Pip(Is)]×♀ LR	0.000±0.000 (>3500; 31)^b^ †	0.000±0.000 (>1000; 9)^b^

For each cross, mean hatching rate ± standard error, number of eggs and egg-rafts are reported.

a and b represent statistical groups (Wilcoxon two sided-test with Bonferonni's adjustment for multiple comparisons).

**†:** This cross is the same as shown in Table 2.

### LR[*w*Pip(Is)] males sterilize field females from islands of the SWIO

LR[*w*Pip(Is)] males were crossed with field females from five populations: Samuel (La Réunion; n = 75 females), Salines (Mauritius; n = 37), Tsoundzou (Mayotte; n = 75) Mada (Madagascar; n = 44) and Grande Glorieuse (Grande Glorieuse; n = 97 females). All crosses were incompatible, displaying >99% embryo mortality (Table 4).Thus, LR[*w*Pip(Is)] males express high CI intensity with field females from the four islands, as observed with females of the LR line.

**Table 4:** Reciprocal crosses between LR[*w*Pip(Is)] line and field specimens.

Crosses	Hatching rate	Outcomes
♂ LR[*w*Pip(Is)]×♀ Samuel	0.011±0.006 (>9000; 75)^a^	bidirectional CI
♂ Samuel×♀ LR[*w*Pip(Is)]	0.000±0.000 (>4400; 36)^a^	
♂LR[*w*Pip(Is)]×♀ Salines	0.007±0.005 (>6000; 51)^a^	bidirectional CI
♂ Salines×♀ LR[*w*Pip(Is)]	0.000±0.000 (>4500; 37)^a^	
♂LR[*w*Pip(Is)]×♀ Grande Glorieuse	0.000±0.000 (>12000; 97)^a^	bidirectional CI
♂ Grande Glorieuse×♀ LR[*w*Pip(Is)]	0.000±0.000 (>500; 40)^a^	
♂LR[*w*Pip(Is)]×♀ Tsoundzou	0.000±0.000 (>9000; 75)^a^	uni- and bidirectional CI
♂ Tsoundzou×♀ LR[*w*Pip(Is)]	0.112±0.092 (>2000; 16)^b^	
♂LR[*w*Pip(Is)]×♀ Mada	0.000±0.000 (>5000; 44)^a^	uni- and bidirectional CI
♂ Mada×♀ LR[*w*Pip(Is)]	0.804±0.283 (>2500; 20)^c^	

Field specimens are from Samuel (La Réunion), Salines (Mauritius), Grande Glorieuse, Tsoundzou (Mayotte) and Mada (Madagascar). For each cross, mean hatching rate ± standard error, number of eggs and egg-rafts are reported.

a, b and c represent statistical groups (Wilcoxon two sided-test with Bonferonni's adjustment for multiple comparisons). Note that, in the cross ♂ Tsoundzou×♀ LR[*w*Pip(Is)], 14 males induced complete CI while 2 were compatible; in the cross ♂ Mada×♀ LR[*w*Pip(Is)], 2 males induced complete CI and 18 were compatible (see text for more details).

Crossing relationships between LR[*w*Pip(Is)] females and field males were also investigated to determine how the LR[*w*Pip(Is)] line may evolve in *Cx. p. quinquefasciatus* field populations in the case of accidental release of LR[*w*Pip(Is)] females. LR[*w*Pip(Is)] females were incompatible with all males from Samuel (n = 36 males), Salines (n = 37) and Grande Glorieuse (n = 40) (Table 4). This shows that LR[*w*Pip(Is)] expresses bidirectional CI with field specimens from these populations. However, males from Tsoundzou (n = 16) were polymorphic for their CI properties, the majority (n = 14) expressing complete CI with LR[*w*Pip(Is)] females and a few (n = 2) being compatible (HR = 0.895±0.035) (Table 4). This shows that LR[*w*Pip(Is)] expresses either bidirectional CI or unidirectional CI with field specimens from Tsoundzou. Thus, two crossing types coexist in Mayotte, but it is likely that the bidirectional CI crossing type is the most frequent one. Males from Mada were also polymorphic for their CI properties but, in contrast to Tsoundzou males, most Mada males were compatible with LR[*w*Pip(Is)] females (n = 18, HR = 0.804±0.283) while only two males expressed CI. So the unidirectional CI type was the most frequent in the Mada population.

### LR[*w*Pip(Is)] and LR males show similar mating performances

**Figure 2:**
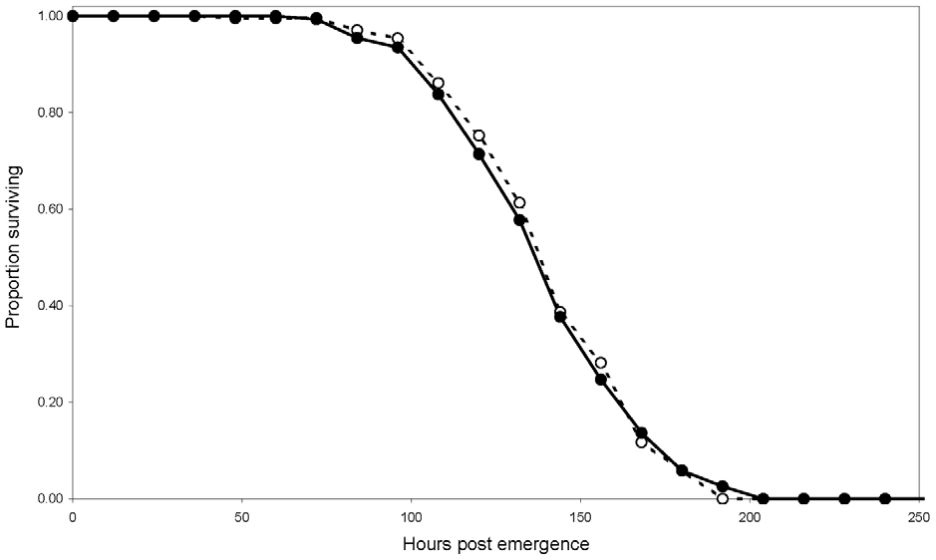
Survival curves for LR (n = 238; dotted line) and LR[*w*Pip(Is)] males (n = 154; solid line).

**Figure 3:**
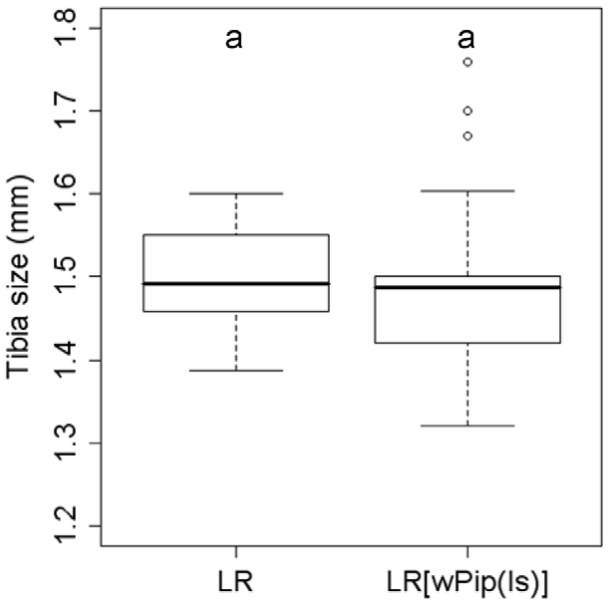
Tibia size of the LR and LR[*w*Pip(Is)] males. Thirty individuals have been measured for each line. ^a^ represents statistical group (Wilcoxon two sided-test).

Inferior competitive ability of LR[*w*Pip(Is)] males compared with field males may limit the efficiency of an IIT program. Thus, the performances of LR[*w*Pip(Is)] and LR males, reared in standardized conditions, were examined for different life history traits. Longevity of LR[*w*Pip(Is)] and LR males (n = 154 and n = 238, respectively) was investigated in conditions where males had to survive by metabolizing nutritional reserves accumulated during their larval life (see material and methods) [69]. No significant difference was found (*χ* ^2^ = 0.04, *P* = 0.84; Figure 2), suggesting that the infection by *w*Pip(Is) did not alter mosquito metabolism. There was also no significant difference between LR[*w*Pip(Is)] and LR male tibia length (n = 30 and n = 30; Wilcoxon two-sided test, *P* = 0.34; Figure 3), a parameter known to be positively correlated with mosquitoes' adult size and reproductive success [70]. This suggests that LR[*w*Pip(Is)] and LR males most probably exhibit similar mating performance.

To further test this assumption, mating competition between LR[*w*Pip(Is)] and LR males was investigated in laboratory cages. Four cages containing different ratios of LR females to LR males to LR[*w*Pip(Is)] males (1∶1∶0, 1∶1∶1, 1∶1∶5 and 1∶1∶10) were set up. Note that as CI occurring between LR[*w*Pip(Is)] males and LR females is complete, it was easy to distinguish egg-rafts produced from compatible (LR males×LR females) or incompatible (LR[*w*Pip(Is)] males×LR females) crosses. Two successive collections of egg-rafts were obtained for each cage by giving females two distinct blood meals. There was no significant variation in the proportion of incompatible egg-rafts between the first and the second series of egg-rafts (Fisher exact test, all *P*>0.57).

As expected, when only LR males were present, all the egg-rafts were compatible (Table 5). In the other cages, no significant difference between LR[*w*Pip(Is)] and LR males' mating capacity was found. Indeed, the number of incompatible egg-rafts observed was not significantly different from expected values assuming an equal competitiveness of LR[*w*Pip(Is)] and LR males and random mating (Binomial test, all *P*>0.18; Table 5). For instance, with an identical ratio of LR[*w*Pip(Is)] and LR males (1∶1), ca. 50% of the egg-rafts produced by LR females were incompatible. When the LR[*w*Pip(Is)] males' ratio was higher than that of LR males, i.e. at 1∶5 and at 1∶10, we observed ca. five and ten times more incompatible egg-rafts than compatible ones. Taken together, these results showed that LR[*w*Pip(Is)] males are as fit as LR males, at least in our laboratory conditions. These experiments also established that LR females cannot discriminate between compatible LR males and incompatible LR[*w*Pip(Is)] males, a result consistent with previous observations of random mating between *Cx. pipiens* mosquitoes infected by incompatible *Wolbachia* strains [37], [48], [71].

**Table 5:** Competition cages with different ratio of LR[*w*Pip(Is)] males.

LR♀∶LR♂∶ LR[wPip(Is)]♂ ratio	Number of adults (number of LR♀, LR♂, LR[*w*Pip(Is)]♂)	Number of egg-rafts (number of eggs)	Observed frequency of infertile egg-rafts (n)	Expected frequency of infertile egg-rafts	P-value*
First blood meal					
1∶1∶0	200 (100, 100, 0)	72 (>7500)	0.00 (72)	0.00	0.99
1∶1∶1	300 (100, 100, 100)	90 (>9000)	0.52 (47)	0.50	0.75
1∶1∶5	350 (50, 50, 250)	43 (>4500)	0.91 (39)	0.83	0.22
1∶1∶10	600 (50, 50, 500)	45 (>4600)	0.98 (44)	0.91	0.18
Second blood meal					
1∶1∶0	_	38 (>7500)	0.00 (38)	0.00	0.99
1∶1∶1	_	42 (>9000)	0.45 (19)	0.50	0.64
1∶1∶5	_	12 (>4500)	0.92 (11)	0.83	0.70
1∶1∶10	_	14 (>4600)	1.00 (14)	0.91	0.63

*: , comparisons between the observed and expected frequencies of infertile egg-rafts through exact binomial test.

## Discussion

The recent expansion of the Rift Valley Fever (RVF) virus [34], [35] combined with high frequencies of insecticide resistance genes in *Cx. p. quinquefasciatus* populations in the SWIO [72] encourage the development of new research to reduce mosquito population densities. Among these approaches, the most promising is the use of *Wolbachia* in an ‘Incompatible Insect Technique’ (IIT), a species-specific control approach in which inundative releases of incompatible males sterilize field *Cx. p. quinquefasciatus* females and possibly lead to the reduction of mosquito population densities.

The present study was undertaken to explore the feasibility of the IIT strategy on the islands of SWIO. We first acquired genetic diversity of *w*Pip strains infecting *Cx. p. quinquefasciatus* mosquitoes from five islands including La Réunion, Mauritius, Mayotte, Madagascar and Grande Glorieuse. All *w*Pip strains from these islands are genetically closely related, belonging to the *w*Pip-I group, which indicates that the *w*Pip diversity is relatively low over this region. However, the variability of crossing types found in Mayotte and Madagascar shows that genetically close *Wolbachia* strains can exhibit distinct CI properties, as observed in some *Cx. pipiens* populations [43], [52] and also in *Drosophila spp.* [73].

Next, we constructed a *Cx. p. quinquefasciatus* LR[*w*Pip(Is)] line that is stably infected with the *w*Pip(Is) strain – a *w*Pip strain previously known to induce bi-directional CI with most La Réunion *w*Pip strains [43]. Care was taken to have a nuclear genetic variability of this line as representative of La Réunion populations as possible. LR[*w*Pip(Is)] males were shown to be as competitive as LR males infected by the native *w*Pip strain, and aging did not affect their CI properties. Finally, the sterilizing capacity of LR[*w*Pip(Is)] males was tested with field females from each of the five islands: LR[*w*Pip(Is)] males were incompatible with all tested field females with very high embryonic mortality (>99%). Overall, these findings demonstrate the feasibility of an IIT program using LR[*w*Pip(Is)] males and encourage field tests for a *Cx. p. quinquefasciatus* elimination strategy in islands of the Indian Ocean.

The geographical isolation of the four islands is an attractive situation for developing an IIT strategy: they are at least 170 km apart from one another and more than 400 km from continental Africa. Thus natural migration is quite unlikely to occur, which should facilitate a local control approach, and minimize the reestablishment of mosquito populations as long as suitable measures are taken for controlling introductions through commercial transport (ships and airplanes, see [74]). Another positive aspect is the small size of La Réunion (2,511 km^2^), Mauritius (2,040 km^2^), Mayotte (374 km^2^) and Grande Glorieuse (7 km^2^), facilitating an exhaustive follow-up of an IIT strategy; this will obviously not be possible on Madagascar because of its size (587,000 km^2^).

However, it must be noted that the success of an IIT strategy could be affected by the accidental release of LR[*w*Pip(Is)] females which might lead to *w*Pip(Is) fixation in natural populations [1], [3], [75]. An efficient sexing system producing only LR[*w*Pip(Is)] males is thus required. Several methods including biological, genetic and transgenic methods have been developed for sex separation of insects [20], [76]. For instance, a biological method consisting of visual separation has been used to hand-select *Cx. p. quinquefasciatus* males [48], but this method is of very limited interest in the context of the large numbers of males needed. Concerning genetic methods, a sex ratio distorter allele, linked to the dominant male-determining gene, has been described in *Cx. pipiens* [77] leading to >80% males in broods. For transgenic methods, a sexing strategy based on the use of Y-linked transgenes expressing fluorescent proteins may be considered, as shown for sexing larvae and pupae in the medfly *Ceratitis capitata* [78]. However, while distorter alleles or transgenes should maximize the production of males for releases, it remains to verify that they do not alter male fitness. An alternative method is the combination of irradiation with CI. Although several studies showed that irradiation can affect male fitness, this scheme was recently tested on *Ae. polynesiensis* by [79] who determined an irradiation dose sufficient to cause sterility of females without sterilising the males or harming their fitness.

In this paper, we present a simple diagnostic PCR based test to genotype *w*Pip infections using the *ank2* marker that could be used to regularly monitor the accidental introduction of the *w*Pip(Is) strain in wild populations. Such a presence would be monitored by analysing *w*Pip strain diversity in mixtures of larvae from natural breeding sites. In the case of the presence of field *w*Pip(Is)-infected individuals, LR[*w*Pip(Is)] male releases would have to be suspended until the elimination of *w*Pip(Is) individuals in the controlled area. Indeed, the bidirectional CI between LR[*w*Pip(Is)] line and field mosquitoes will prevent the establishment of *w*Pip(Is) infected individuals in these islands.

### Conclusion

The study presented here supports the feasibility of an IIT strategy using the LR[*w*Pip(Is)] males and targeting field *Cx. p. quinquefasciatus* populations, a species of medical and veterinary concern in the SWIO islands. This method now needs to be further tested in semi-field conditions in order to optimize several key parameters, i.e. the number of males to be released as well as the timing of releases. Recently, new semi-field cages were developed to measure the impact of the life-shortening *Wolbachia w*MelPop strain on populations of *Aedes aegypti* [80]. Such cages provide a realistic transitional platform between laboratory and field conditions. The risk of accidental releases of females needs also to be limited by developing an efficient sexing method to prevent any unintentional *Wolbachia* replacement.

## Supporting Information

Figure S1 **Backcrossing procedure.** Mosquito nuclear backgrounds are indicated by colours: black represents *Cx. p. quinquefasciatus* nuclear background (LR and LR-TC lines) and red represents *Cx. p. pipiens* nuclear background (Is line). *Wolbachia* infection types are indicated by w-labelled symbols: black-filled symbols represent the *w*Pip(LR) strain and red-filled symbols the *w*Pip(Is) strain. Note that the LR[*w*Pip(Is)] line carries the LR nuclear background and the *w*Pip(Is) infection and could be used to produce incompatible males for field release; LR-TC is an uninfected mosquito line.
(TIF)

Figure S2 **Genetic patterns of** ***Culex pipiens*** **lines and their** ***Wolbachia*** **strains.** A, PCR-RFLP of the *Cx. pipiens ace-2* gene digested by *ScaI* enzyme; B, PCR-RFLP of the *Cx. pipiens Ester^2^* gene digested by *AvaII* enzyme; C, PCR products of the *Wolbachia ank2* gene. The LR[*w*Pip(Is)] line carries the LR nuclear background and the *w*Pip(Is) infection; LR-TC is an uninfected mosquito line. M, molecular weight markers; kb, kilo bases.
(TIF)

Table S1 **Genes and primers of** ***Wolbachia*** **and** ***Culex pipiens*** **.**
(DOC)

## References

[1] Dobson SL (2003). Reversing *Wolbachia*-based population replacement.. Trends Parasitol.

[2] Sinkins SP, Gould F (2006). Gene drive systems for insect disease vectors.. Nat Rev Genet.

[3] Bourtzis K (2008). *Wolbachia*-based technologies for insect pest population control.. Adv Exp Med Biol.

[4] Saridaki A, Bourtzis K (2010). *Wolbachia*: more than just a bug in insect genitals.. Curr Opin Microbiol.

[5] Cook PE, McGraw EA (2010). *Wolbachia pipientis*: an expanding bag of tricks to explore for disease control.. Trends Parasitol.

[6] Werren JH, Windsor DM (2000). *Wolbachia* infection frequencies in insects: evidence of a global equilibrium?. Proc R Soc Lond B.

[7] Duron O, Bouchon D, Boutin S, Bellamy L, Zhou L (2008). The diversity of reproductive parasites among arthropods: *Wolbachia* do not walk alone.. BMC Biology.

[8] Hilgenboecker K, Hammerstein P, Schlattmann P, Telschow A, Werren JH (2008). How many species are infected with *Wolbachia*? A statistical analysis of current data.. FEMS Microbiol Lett.

[9] Yen JH, Barr AR (1971). New hypothesis of the cause of cytoplasmic incompatibility in *Culex pipiens* L.. Nature.

[10] Rasgon JL, Scott TW (2003). *Wolbachia* and cytoplasmic incompatibility in the California *Culex pipiens* mosquito species complex: parameter estimates and infection dynamics in natural populations.. Genetics.

[11] O'Neill SL, Giordano R, Colbert AM, Karr TL, Robertson HM (1992). 16S rRNA phylogenetic analysis of the bacterial endosymbionts associated with cytoplasmic incompatibility in insects.. Proc Natl Acad Sci USA.

[12] Xi Z, Khoo CC, Dobson SL (2005). *Wolbachia* establishment and invasion in an *Aedes aegypti* laboratory population.. Science.

[13] Ruang-Areerate T, Kittayapong P (2006). *Wolbachia* transinfection in *Aedes aegypti*: a potential gene driver of dengue vectors.. Proc Natl Acad Sci USA.

[14] McMeniman CJ, Lane RV, Cass BN, Fong AW, Sidhu M (2009). Stable introduction of a life-shortening *Wolbachia* infection into the mosquito *Aedes aegypti*.. Science.

[15] Werren JH, Baldo L, Clark ME (2008). *Wolbachia*: master manipulators of invertebrate biology.. Nat Rev Microbiol.

[16] Engelstadter J, Hurst GDD (2009). The ecology and evolution of microbes that manipulate host reproduction.. Annu Rev Ecol Evol Syst.

[17] Serbus LR, Casper-Lindley C, Landmann F, Sullivan W (2008). The genetics and cell biology of *Wolbachia*-host interactions.. Annu Rev Genet.

[18] Dobson SL, Fox CW, Jiggins FM (2002). The effect of *Wolbachia*-induced cytoplasmic incompatibility on host population size in natural and manipulated systems.. Proc R Soc Lond B.

[19] Zabalou S, Riegler M, Theodorakopoulou M, Stauffer C, Savakis C (2004). *Wolbachia*-induced cytoplasmic incompatibility as a means for insect pest population control.. Proc Natl Acad Sci USA.

[20] Zabalou S, Apostolaki A, Livadaras I, Franz G, Robinson AS (2009). Incompatible insect technique: incompatible males from a *Ceratitis capitata* genetic sexing strain.. Ent Exp Appl.

[21] Calvitti M, Moretti R, Lampazzi E, Bellini R, Dobson SL (2010). Characterization of a new *Aedes albopictus* (Diptera: Culicidae)-*Wolbachia pipientis* (Rickettsiales: Rickettsiaceae) symbiotic association generated by artificial transfer of the *w*Pip strain from *Culex pipiens* (Diptera: Culicidae).. J Med Entomol.

[22] Apostolaki A, Livadaras I, Saridaki A, Chrysargyris A, Savakis C (2011). Transinfection of the olive fruit fly Bactrocera oleae with *Wolbachia*: towards a symbiont-based population control strategy.. J Appl Entomol.

[23] Rasgon JL, Scott TW (2004). Impact of population age structure on *Wolbachia* transgene driver efficacy: ecologically complex factors and release of genetically modified mosquitoes.. Insect Biochem Mol Biol.

[24] Moreira LA, Iturbe-Ormaetxe I, Jeffery JA, Lu G, Pyke AT (2009). A *Wolbachia* symbiont in *Aedes aegypti* limits infection with dengue, Chikungunya, and Plasmodium.. Cell.

[25] Kambris Z, Cook PE, Phuc HK, Sinkins SP (2009). Immune activation by life-shortening *Wolbachia* and reduced filarial competence in mosquitoes.. Science.

[26] Kambris Z, Blagborough AM, Pinto SB, Blagrove MS, Godfray HC (2010). *Wolbachia* stimulates immune gene expression and inhibits plasmodium development in *Anopheles gambiae*.. PLoS Pathog.

[27] Barr AR, Tabachnik WJ, Rai KS, Narang S (1982). The *Culex pipiens* complex.. Recent developments in the genetics of insect disease vectors, Steiner WWM.

[28] Sabatinelli G, Ranieri E, Gianzi FP, Papakay M, Cancrini G (1994). Role of *Culex quinquefasciatus* in the transmission of bancroftian filariasis in the Federal Islamic Republic of Comoros (Indian Ocean).. Parasite.

[29] Hemingway J, Ranson H (2000). Insecticide resistance in insect vectors of human disease.. Annu Rev Entomol.

[30] Charrel RN, de Lamballerie X (2004). West Nile virus, an emerging arbovirus.. Presse Med.

[31] Durand JP, Simon F, Tolou H (2004). West Nile virus: in France again, in humans and horses.. Rev Prat.

[32] Komar N (2003). West Nile virus: epidemiology and ecology in North America.. Adv Virus Res.

[33] Granwehr BP, Lillibridge KM, Higgs S, Mason PW, Aronson JF (2004). West Nile virus: where are we now?. Lancet Infect Dis.

[34] Sissoko D, Giry C, Gabrié P, Tarantola A, Pettinelli F (2009). Rift Valley fever, Mayotte, 2007–2008.. Emerg Infect Dis.

[35] Roger M (2011). Rift Valley Fever in ruminants, Republic of Comoros, 2009.. Emerg Infect Dis.

[36] Duron O, Lagnel J, Raymond M, Bourtzis K, Fort P (2005). Transposable element polymorphism of *Wolbachia* in the mosquito *Culex pipiens*: evidence of genetic diversity, superinfection and recombination.. Mol Ecol.

[37] Duron O, Raymond M, Weill M (2011). Many compatible *Wolbachia* strains coexist within natural populations of *Culex pipiens* mosquito.. Heredity.

[38] Atyame CM, Delsuc F, Pasteur N, Weill M, Duron O (2011). Diversification of *Wolbachia* endosymbiont in the *Culex pipiens* mosquito.. Mol Biol Evol.

[39] Laven H, Wright J, Pal R (1967). Speciation and evolution in *Culex pipiens*.. Genetics of Insect Vectors of Disease.

[40] O'Neill SL, Paterson HE (1992). Crossing type variability associated with cytoplasmic incompatibility in Australian populations of the mosquito *Culex quinquefasciatus* Say.. Med Vet Entomol.

[41] Guillemaud T, Pasteur N, Rousset F (1997). Contrasting levels of variability between cytoplasmic genomes and incompatibility types in the mosquito *Culex pipiens*.. Proc R Soc Lond B.

[42] Duron O, Bernard C, Unal S, Berthomieu A, Berticat C (2006). Tracking factors modulating cytoplasmic incompatibilities in the mosquito *Culex pipiens*.. Mol.

[43] Atyame CM, Duron O, Tortosa P, Pasteur N, Fort P (2011). Multiple *Wolbachia* determinants control the evolution of cytoplasmic incompatibilities in *Culex pipiens* mosquito populations.. Mol Ecol.

[44] Lindquist DA, Abusowa M, Hall MJ (1992). The New World screwworm fly in Libya: a review of its introduction and eradication.. Med Vet Entomol.

[45] Alphey L, Benedict M, Bellini R, Clark GG, Dame DA (2010). Sterile-insect methods for control of mosquito-borne diseases: an analysis.. Vector Borne Zoonotic Dis.

[46] Collins SR, Weldon CW, Banos C, Taylor PW (2008). Effects of irradiation dose rate on quality and sterility of Queensland fruit flies, *Bactrocera tryoni* (Froggatt).. Journal of Applied Entomology.

[47] Kumano N, Haraguchi D, Kohama T (2008). Effect of irradiation on mating performance and mating ability in the West Indian sweetpotato weevil, *Euscepes postfasciatus*.. Entomologia Experimentalis et Applicata.

[48] Laven H (1967). Eradication of *Culex pipiens fatigans* through cytoplasmic incompatibility.. Nature.

[49] Brelsfoard CL, Sechan Y, Dobson SL (2008). Interspecific hybridization yields strategy for South pacific filariasis vector elimination.. PLoS Negl Trop Dis.

[50] Duron O, Fort P, Weill M (2006). Hypervariable prophage WO sequences describe an unexpected high number of *Wolbachia* variants in the mosquito *Culex pipiens*.. Proc R Soc Lond B.

[51] Rogers SO, Bendich AJ, Gelvin SB, Schilperoort RA (1988). Extraction of DNA from plant tissues.. Plant Molecular Biology Manuel, Volume A6.

[52] Duron O, Boureux A, Echaubard P, Berthomieu A, Berticat C (2007). Variability and expression of ankyrin domain genes in *Wolbachia* variants infecting the mosquito *Culex pipiens*.. J Bacteriol.

[53] Malcolm CA, Bourguet D, Ascolillo A, Rooker SJ, Garvey CF (1998). A sex-linked *Ace* gene, not linked to insensitive acetylcholinesterase-mediated insecticide resistance in *Culex pipiens*.. Insect Mol Biol.

[54] Raymond M, Poulin E, Boiroux V, Dupont E, Pasteur N (1993). Stability of insecticide resistance due to amplification of esterase genes in *Culex pipiens*.. Heredity.

[55] Bourguet D, Foncesca D, Vourch G, Dubois MP, Chandre F (1998). The acetylcholinesterase gene ace: a diagnostic marker of the *pipiens* and *quinquefasciatus* forms of the *Culex pipiens* complex.. J Amer Mosq Control Assoc.

[56] Kumar S, Tamura K, Nei M (2004). MEGA3: Integrated software for molecular evolutionary genetics analysis and sequence alignment.. Briefings in Bioinformatics.

[57] Duron O, Weill M (2006). *Wolbachia* infection influences the development of *Culex pipiens* embryo in incompatible crosses.. Heredity.

[58] Crawley M (2007). The R Book..

[59] Boyle L, O'Neill SL, Robertson HM, Karr TL (1993). Interspecific and intraspecific horizontal transfer of *Wolbachia* in *Drosophila*.. Science.

[60] Poinsot D, Bourtzis K, Markakis G, Savakis C, Mercot H (1998). *Wolbachia* transfer from *Drosophila melanogaster* into *D. simulans*: Host effect and cytoplasmic incompatibility relationships.. Genetics.

[61] Hornett EA, Duplouy AM, Davies N, Roderick GK, Wedell N (2008). You can't keep a good parasite down: evolution of a male-killer suppressor uncovers cytoplasmic incompatibility.. Evolution.

[62] Walker T, Song S, Sinkins SP (2009). *Wolbachia* in the *Culex pipiens* group mosquitoes: Introgression and superinfection.. Journal of Heredity.

[63] Singh KRP, Curtis CF, Krishnamurthy BS (1976). Partial loss of cytoplasmic incompatibility with age in males of *Culex fatigans*.. Ann Trop Med Parasit.

[64] Hoffmann AA, Turelli M, Harshman LG (1990). Factors affecting the distribution of cytoplasmic incompatibility in *Drosophila simulans*.. Genetics.

[65] Turelli M, Hoffmann AA (1995). Cytoplasmic incompatibility in *Drosophila simulans*: dynamics and parameter estimates from natural populations.. Genetics.

[66] Jamnongluk W, Kittayapong P, Baisley KJ, O'Neill SL (2000). *Wolbachia* infection and expression of cytoplasmic incompatibility in *Armigeres subalbatus* (Diptera: Culicidae).. J Med Entomol.

[67] Kittayapong P, Mongkalangoon P, Baimai V, O'Neill SL (2002). Host age effect and expression of cytoplasmic incompatibility in field populations of *Wolbachia*-superinfected *Aedes albopictus*.. Heredity.

[68] Duron O, Fort P, Weil M (2007). Influence of aging on cytoplasmic incompatibility, sperm modification and *Wolbachia* density in *Culex pipiens* mosquitoes.. Heredity.

[69] Briegel H (1990). Metabolic relationship between female body size, reserves, and fecundity of *Aedes aegypti*.. Journal of Insect Physiology.

[70] Clements AN (1992).

[71] Curtis CF, Brooks GD, Ansari MA, Grover KK, Krishnamurthy BS (1982). A field trial on control of *Culex quinquefasciatus* by release of males of a strain integrating cytoplasmic incompatibility and a translocation.. Ent Exp Appl.

[72] Tantely ML, Tortosa P, Alout H, Berticat C, Berthomieu A (2010). Insecticide resistance in *Culex pipiens quinquefasciatus* and *Aedes albopictus* mosquitoes from La Reunion Island.. Insect Biochem Mol Biol.

[73] Zabalou S, Apostolaki A, Pattas S, Veneti Z, Paraskevopoulos C (2008). Multiple rescue factors within a *Wolbachia* strain.. Genetics.

[74] Raymond M, Callaghan A, Fort P, Pasteur N (1991). Worldwide migration of amplified insecticide resistance genes in mosquitoes.. Nature.

[75] Bourtzis K, Robinson AS, Bourtzis K, Miller T (2006). Insect pest control using *Wolbachia* and/or radiation.. Insect Symbiosis 2.

[76] Papathanos PA, Bossin HC, Benedict MQ, Catteruccia F, Malcolm CA (2009). Sex separation strategies: past experience and new approaches.. Malar J.

[77] Sweeny TL, Barr AR (1978). Sex ratio distortion caused by meiotic drive in a mosquito, *Culex pipiens* L.. Genetics.

[78] Condon KC, Condon GC, Dafa'alla TH, Fu G, Phillips CE (2007). Genetic sexing through the use of Y-linked transgenes.. Insect Biochem Mol Biol.

[79] Brelsfoard CL, St Clair W, Dobson SL (2009). Integration of irradiation with cytoplasmic incompatibility to facilitate a lymphatic filariasis vector elimination approach.. Parasit Vectors.

[80] Ritchie SA, Johnson PH, Freeman AJ, Odell RG, Graham N (2011). A secure semi-field system for the study of *Aedes aegypti*.. PLoS Negl Trop Dis.

