# Supplementary figures and images for: Cytoplasmic Incompatibility as a Means of Controlling Culex pipiens quinquefasciatus Mosquito in the Islands of the South-Western Indian Ocean
Source: PLoS Negl Trop Dis. 2011 Dec 20;5(12):e1440. doi: 10.1371/journal.pntd.0001440 (PMC3243720; doi:10.1371/journal.pntd.0001440)

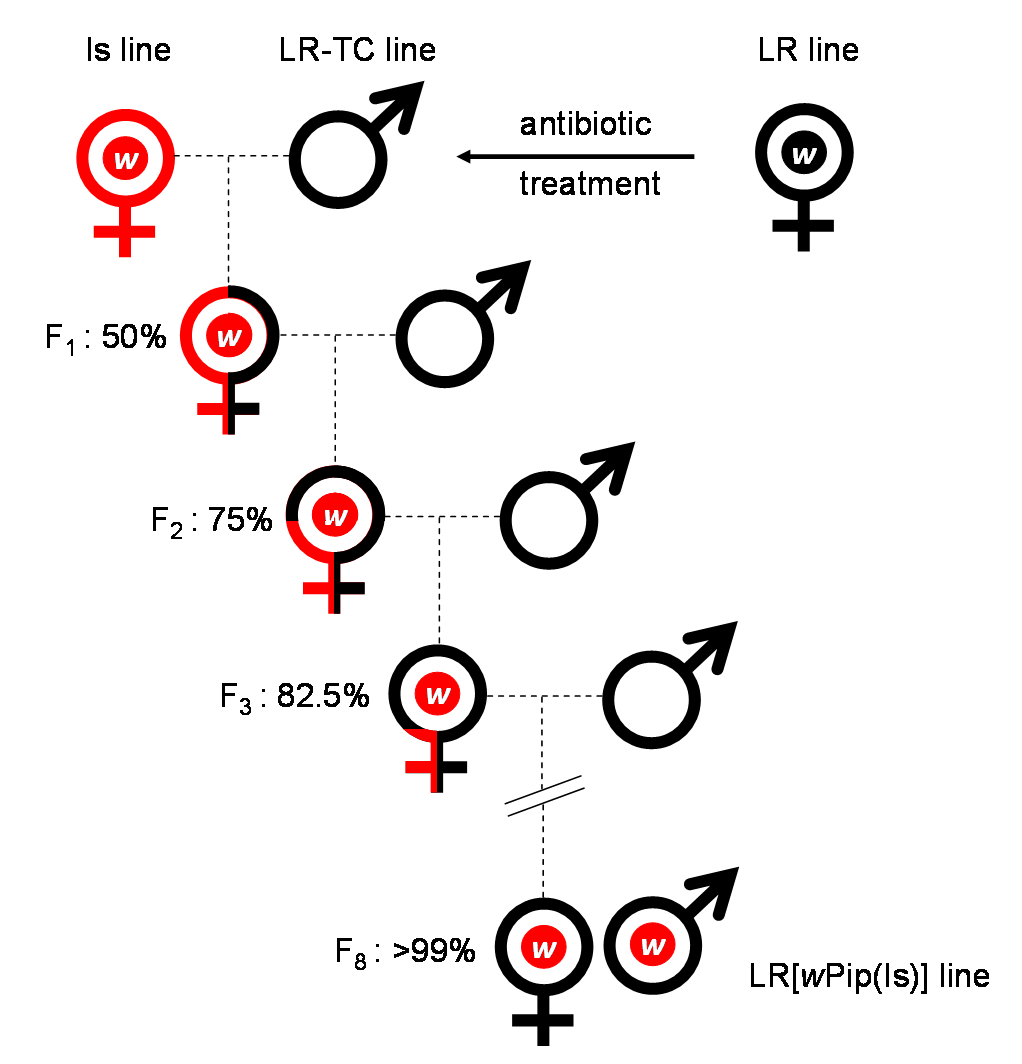

Supplement: Figure S1 — Backcrossing procedure. Mosquito nuclear backgrounds are indicated by colours: black represents Cx. p. quinquefasciatus nuclear background (LR and LR-TC lines) and red represents Cx. p. pipiens nuclear background (Is line). Wolbachia infection types are indicated by w-labelled symbols: black-filled symbols represent the wPip(LR) strain and red-filled symbols the wPip(Is) strain. Note that the LR[wPip(Is)] line carries the LR nuclear background and the wPip(Is) infection and could be used to produce incompatible males for field release; LR-TC is an uninfected mosquito line. (TIF) [file pntd.0001440.s001.tif]

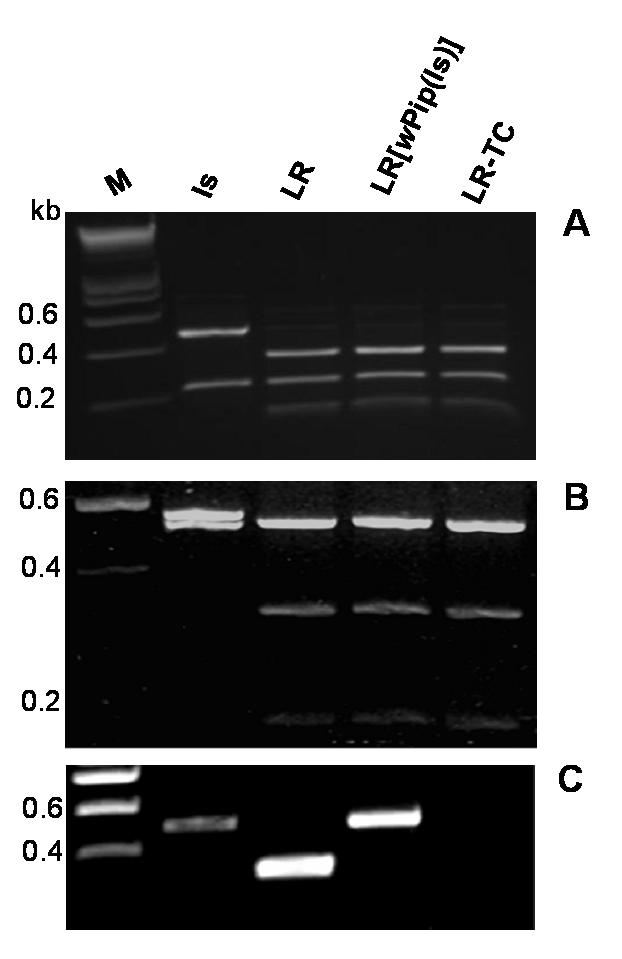

Supplement: Figure S2 — Genetic patterns of Culex pipiens lines and their Wolbachia strains. A, PCR-RFLP of the Cx. pipiens ace-2 gene digested by ScaI enzyme; B, PCR-RFLP of the Cx. pipiens Ester2 gene digested by AvaII enzyme; C, PCR products of the Wolbachia ank2 gene. The LR[wPip(Is)] line carries the LR nuclear background and the wPip(Is) infection; LR-TC is an uninfected mosquito line. M, molecular weight markers; kb, kilo bases. (TIF) [file pntd.0001440.s002.tif]
