# Supplementary material for: Cytoplasmic Incompatibility as a Means of Controlling Culex pipiens quinquefasciatus Mosquito in the Islands of the South-Western Indian Ocean
Source: PLoS Negl Trop Dis. 2011 Dec 20;5(12):e1440. doi: 10.1371/journal.pntd.0001440 (PMC3243720; doi:10.1371/journal.pntd.0001440)
Supplement: Table S1 — Genes and primers of Wolbachia and Culex pipiens . (DOC) [file pntd.0001440.s003.doc]

| Gene | | Putative product | Primer (5'-3') | Size (bp) | References |
| --- | --- | --- | --- | --- | --- |
| *Wolbachia* gene | | |  |  |  |
|  | *ank2* | Ankyrin domain protein | F-CTTCTTCTGTGAGTGTACGT | 313-511 | [1] |
|  |  |  | R2-TCCATATCGATCTACTGCGT |  |  |
| *Culex pipiens* genes | | |  |  |  |
|  | *ace-2* | Acetylcholinesterase 2 (AChE2) | F1457-GAGGAGATGTGGAATCCCAA | 700 | [2] |
|  |  |  | B1246-TGGAGCCTCCTCTTCACGGC |  |  |
|  | *Ester^2^* | Carboxylester hydrolase | Bdir1530-CTCCAGATCAACCCTTC | 1100 | [3] |
|  |  |  | MMI_R-CAGCTTCGGGTCGATCATCAT |  |  |

References

1- Duron O, Boureux A, Echaubard P, Berthomieu A, Berticat C, et al. (2007) Variability and expression of ankyrin domain genes in *Wolbachia* variants infecting the mosquito *Culex pipiens.* J Bacteriol 189: 4442-4448.

2- Bourguet D, Foncesca D, Vourch G, Dubois MP, Chandre F, et al. (1998) The acetylcholinesterase gene ace: a diagnostic marker of the pipiens and quinquefasciatus forms of the Culex pipiens complex. J. Amer. Mosq. Control Assoc. 14: 390-396.

3- Ben Cheikh R, Berticat C, Berthomieu A, Pasteur N, Ben Cheikh H, Weill M. (2008) Characterization of a novel high-activity esterase in Tunisian Populations of the mosquito Culex pipiens. Journal of Economic Entomology 101: 484-491.
